# A signature for success

**DOI:** 10.7554/eLife.08773

**Published:** 2015-06-16

**Authors:** Joaquín M Espinosa, Kelly D Sullivan

**Affiliations:** Howard Hughes Medical Institute and Department of Molecular, Cellular and Developmental Biology, University of Colorado at Boulder, Boulder, United Statesjoaquin.espinosa@colorado.edu; Howard Hughes Medical Institute and Department of Molecular, Cellular and Developmental Biology, University of Colorado at Boulder, Boulder, United States

**Keywords:** translational oncology, predictive signature, p53, human

## Abstract

The expression pattern of 13 genes can predict whether cancer cells will be sensitive to drugs that inhibit a protein that represses the activity of p53.

**Related research article** Jeay S, Gaulis S, Ferretti S, Bitter H, Ito M, Valat T, Murakami M, Ruetz S, Guthy DA, Rynn C, Jensen MR, Wiesmann M, Kallen J, Furet P, Gessier F, Holzer P, Masuya K, Würthner J, Halilovic E, Hofmann F, Sellers WR, Porta DG. 2015. A distinct p53 target gene set predicts for response to the selective p53-HDM2 inhibitor NVP-CGM097. *eLife* **4**:e06498*.* doi: 10.7554/eLife.06498

**Image** A gene expression ‘signature’ was used to predict whether cancer cell lines would be sensitive (pink) or insensitive (blue) to drug treatment

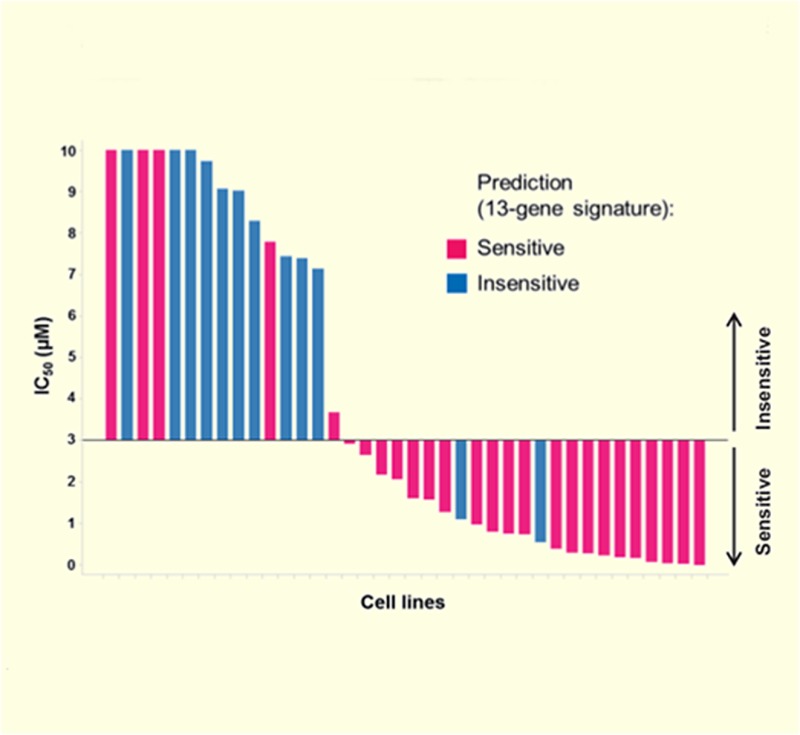

Cancer develops when cells acquire genetic mutations that allow them to divide rapidly and invade neighboring tissues. Currently, most drugs used to treat cancers work by poisoning all the rapidly dividing cells in the body, including cells that are healthy, and this leads to unpleasant side effects. The main goal of personalized cancer medicine is to replace these toxic drugs with therapies that target specific abnormalities within the cells of individual cancers. Unfortunately, most targeted therapies fail in the clinic even when the abnormality they are meant to be targeting is present, which reveals our lack of understanding of how cancer cells respond to these drugs.

Eleven years ago, the first targeted therapies that reactivate a protein called p53, which is inactivated in many cancer cells, were developed. However, in most types of cancer cells tested, restoring activity of the p53 tumor suppressor failed to kill the cancer cells. Now, in *eLife*, Diana Graus Porta and colleagues at the Novartis Institutes for BioMedical Research—including Sebastien Jeay as first author—describe a ‘biomarker’ that can predict whether or not different cancer cells will die if p53 is reactivated (Jeay et al., 2015).

p53 controls programmed cell death (apoptosis), the arrest of cell division and other cell responses to stress by binding to DNA and increasing the expression of particular genes (Bieging et al., 2014). However, despite decades of study, we do not fully understand how cells respond to p53 activity. Around 50% of all human cancers have mutations in *TP53*, the gene that encodes the p53 tumor suppressor protein, and these tumors produce inactive forms of p53. However, in the other cancers, p53 is inactivated in other ways, such as by the over-activation of MDM2 (sometimes known as HDM2), a protein that represses the activity of p53. Therefore, 11 million cancer patients worldwide have tumors with normal p53 proteins that may benefit from treatment with drugs that can inhibit the activity of MDM2.

The first MDM2 inhibitor, called Nutlin-3, was discovered in 2004. Since then, a flurry of activity in the pharmaceutical industry has led to development of several similar compounds, many of which are currently being tested in clinical trials (Khoo et al., 2014). Although these drugs are able to activate p53 and induce changes in the expression of many of the genes involved in apoptosis, this does not lead to increased levels of cell death in most types of cancer cells (Tovar et al., 2006; Garnett et al., 2012). These drugs stop the cells from dividing, but this is only a temporary effect and so is of limited therapeutic value. What factors determine whether or not a cancer cell dies when MDM2 is inhibited?

Jeay et al. describe two new MDM2 inhibitors called NVP-CFC218 and NVP-CGM097. These compounds mimic the interactions between p53 and MDM2 to prevent them from interacting with each other, which results in the activation of p53. Like other inhibitors that target the interactions between p53 and MDM2, both compounds only affect cells that carry normal p53 proteins. Jeay et al. tested the effects of both drugs on the growth of cells in 356 different cancer cell lines, and found that cells in 47 of the lines stopped growing (referred to as sensitive), but cells in 309 of the lines were unaffected (insensitive). Although all of the sensitive lines had normal p53 as expected, the majority of the cell lines with normal p53 were insensitive to these drugs. Therefore, the presence of mutations in the *TP53* gene alone is not a good indicator of the response of cancer cells to these drugs.

To address the need for a better biomarker, Jeay et al. compared the gene expression profiles of the sensitive and insensitive cell lines before the drug treatment. They discovered a gene expression ‘signature’ of 13 genes that can predict the response of cells to treatment with NVP-CFC218 or NVP-CGM097. After confirming the predictive power of this signature in several different cancer cell lines, they tested this biomarker in tumor samples collected from 55 patients. 19 of the 27 of the tumor samples predicted to be sensitive to the drugs decreased in size after treatment, yielding a predictive value of more than 70% (Figure 1).

**Figure 1.**
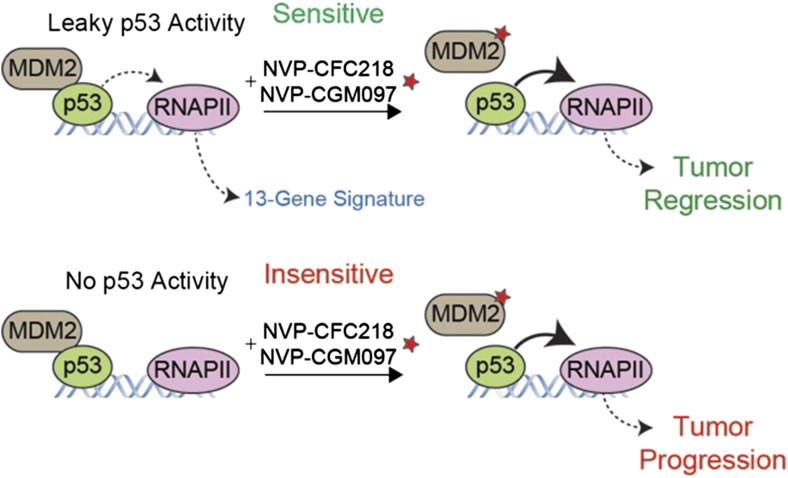
A gene expression signature predicts the response of cells to drug treatments that inhibit MDM2. The tumor suppressor protein p53 promotes cell death and halts cell division by activating an enzyme called RNA polymerase II (RNAPII) to drive the expression of particular genes. However, in many cancer cells, p53 is deactivated by another protein called MDM2 (left). Jeay et al. report that a “signature” formed by the expression levels of 13 genes can predict whether or not cancer cells will respond to two drugs -- NVP-CFC218 and NVP-CGM097 (denoted by a red star) -- that inhibit the activity of MDM2. The signature predicted that 27 of the 55 tumor samples collected from patients would be sensitive to the drugs, and 19 of the 27 tumors decreased in size after treatment (top). The 13 genes in the signature are all regulated by p53, which suggests that there is least some ‘leaky’ p53 activity (indicated by the dotted line) in the cells of sensitive tumors before the drug treatment (top left). The other 36 tumor samples—most of which lacked the gene signature—continued to grow after the drug treatment (bottom), although the reasons for this are not fully understood.

Intriguingly, the gene expression signature is made entirely of genes that are known to be directly regulated by p53, which implies that there is a partially active p53 signaling pathway in the sensitive cells prior to the drug treatment. This finding raises some tantalizing questions: is this p53 activity required for cells to be sensitive, and how is it achieved? Do the sensitive cell lines have a protein that is essential for p53 activity that has been inactivated in the insensitive cells? Do the insensitive cells have another protein that inhibits p53—other than MDM2—that is lost in sensitive cells? Or, is it easier for p53 to access DNA in sensitive cells to promote gene expression? Is the p53 activity in sensitive cells a sign that the cells are experiencing stress prior to the drug treatment? Answering these questions will both advance our understanding of the biology of p53, and also help us to harness the remarkable power of p53 for therapeutic use.

As more targeted therapies are developed, it is absolutely essential that biomarkers accompany them to the market. The Novartis team describes an elegant, yet straightforward method for determining gene expression signatures that can predict the responses of cells to drug treatments, and medical researchers should consider using similar methods during the preclinical phase of the development of all new drugs. Gene expression signatures may also lead to more effective therapies that use combinations of drugs. Hopefully, the discovery of this signature will accelerate the deployment of MDM2 inhibitors into the clinic, which will benefit millions of cancer patients carrying tumors with normal p53 that has been deactivated by MDM2.
